# An integrated view of data quality in Earth observation

**DOI:** 10.1098/rsta.2012.0072

**Published:** 2013-01-28

**Authors:** X. Yang, J. D. Blower, L. Bastin, V. Lush, A. Zabala, J. Masó, D. Cornford, P. Díaz, J. Lumsden

**Affiliations:** 1 Reading e-Science Centre, Environmental Systems Science Centre, University of Reading, Reading RG6 6AL, UK; 2 School of Engineering and Applied Science, Aston University, Birmingham B4 7ET, UK; 3 Department of Geography, Universitat Autònoma de Barcelona, 08193 Bellaterra (Cerdanyola del Vallès), Spain; 4 CREAF, Cerdanyola del Vallès 08193, Spain

**Keywords:** data quality, uncertainty, environmental informatics, Earth observation, provenance, metadata

## Abstract

Data quality is a difficult notion to define precisely, and different communities have different views and understandings of the subject. This causes confusion, a lack of harmonization of data across communities and omission of vital quality information. For some existing data infrastructures, data quality standards cannot address the problem adequately and cannot fulfil all user needs or cover all concepts of data quality. In this study, we discuss some philosophical issues on data quality. We identify actual user needs on data quality, review existing standards and specifications on data quality, and propose an integrated model for data quality in the field of Earth observation (EO). We also propose a practical mechanism for applying the integrated quality information model to a large number of datasets through metadata inheritance. While our data quality management approach is in the domain of EO, we believe that the ideas and methodologies for data quality management can be applied to wider domains and disciplines to facilitate quality-enabled scientific research.

## 1. Introduction

Earth observation (EO) is the science of measurement of all aspects of the Earth system, including its physical, chemical and biological processes. Historically, the term has been applied mostly to satellite-based remote sensing, but modern usage encompasses a much broader field of observing systems, including low-level EO (from aircraft and unmanned aerial vehicles), *in situ* measurements and sampling campaigns. A key characteristic of EO data is that they are very commonly reused many times in different scientific studies and decision-making processes. For example, the digital elevation model (DEM) from the NASA Shuttle Radar Topography Mission (SRTM; [1]) has been reported to have been served to 750 000 users from 221 countries.

The Group on Earth Observations (GEO) is a voluntary partnership of (as of September 2011) 87 governments, the European Commission and 64 intergovernmental, international and regional organizations. GEO is coordinating the construction of the Global Earth Observation System of Systems (GEOSS), an information technology infrastructure that provides access to EO data from a large number of observing systems. The GEOSS contains around 95 000 datasets (as of October 2011) and so it is widely recognized that users need to be provided with information in order to assess a dataset's fitness for their purpose, and to assist them in using the dataset correctly. The communication of data *quality* is therefore very important.

The Quality Assurance for Earth Observation (QA4EO) initiative has produced a number of guidelines that have been adopted as GEOSS ‘best practice’ documents. The key principle of QA4EO is that all data and derived products must have associated with them ‘…a Quality Indicator (QI), which must be unequivocal and universal in terms of its definition and derivation … based on a statistically derived value. This value should be the result of an assessment of its traceability to an agreed reference standard (ideally SI) as propagated through the data processing chain’ [2], p. 3. Quality indicators may be entirely objectively derived by measurement and calculation or, if necessary, may be elicited by subjective expert judgement, but the data provider *must* specify how the quality indicator was derived.

The importance of spatial data quality indicators is widely recognized in the scientific literature [3–5]. Devillers *et al.* [4] argue that quality indicators are ‘a way of seeing the big picture by looking at a small piece of it’. They suggest that quality indicators can inform users of a global measure of quality without them having to examine the data in much detail.

We concur with the QA4EO guidelines and build on them in this paper, addressing the following questions:

— What are the different concepts and uses of quality information (§2)?
— What kinds of quality indicators are required by EO data users (§3)?
— What is the current state of the GEOSS with regard to the provision of quality information (§4)?
— How can we form a new, integrated view of data quality that will allow the GEOSS to provide more complete quality information (§5)?

These studies were performed in the context of the GeoViQua project (http://www.geoviqua.org). GeoViQua considers the full life cycle of data quality information, from elicitation and derivation, through encoding in metadata documents that are linked to data, through to the use of the quality information in search and visualization tasks. This study clarifies concepts of data quality, identifies user needs, reviews associated existing quality standards and specifications, and proposes an expanded quality information model. Actual methods to elicit and evaluate data quality and to derive probability distributions or error estimates are not included in the scope of this particular paper but are being actively researched in the GeoViQua project. Although these studies focus on the science of EO and the GEOSS, the principles and approaches can be readily applied to metrology and data-sharing in other scientific disciplines.

## 2. Data quality concepts

**Figure 1.**
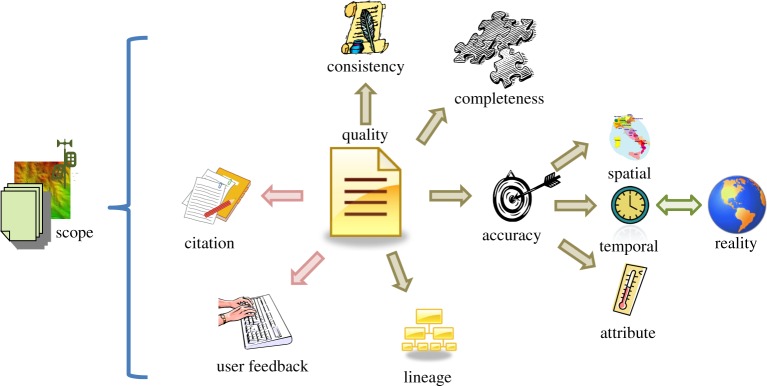
Overview of the key concepts of data quality addressed in this study, including both producer- and consumer-created information. (Online version in colour.)

Data quality is a difficult notion to define precisely. It means different things to different communities. ISO 9000 [6] defines quality as ‘the totality of characteristics of a product that bear on its ability to satisfy stated and implied needs, degree to which a set of inherent characteristics fulfils requirements’. A key challenge for EO is that it is generally impossible to state all actual or implied needs or requirements, because each data item might be used for many purposes, some of which are not foreseeable in advance. Data quality itself has many facets, as illustrated in figure 1.

### (a) Terminology

One source of confusion is that terms with distinct meanings are sometimes incorrectly used interchangeably, particularly the terms *accuracy*, *uncertainty* and *error*.

Accuracy can be defined as the closeness of agreement between an observed value and the true value or, where a true value cannot be obtained, the reference value which is accepted as true.

Uncertainty is the most natural method to quantify accuracy and is defined as ‘the lack of certainty, a state of having limited knowledge where it is impossible to exactly describe existing state or historical state/future outcome’ [7], or ‘the situation where the current state of knowledge is such that the order or nature of things is unknown and the consequences, extent, or magnitude of circumstances, conditions, or events is unpredictable’ [8]. This definition refers largely to uncertainty due to a lack of knowledge, often referred to as *epistemic* uncertainty. There are whole taxonomies of uncertainty [9,10], but these are not considered further at this point.

The statistical *error* can be defined to be ‘the amount by which an observation differs from its (unobservable) true value’. Because the true value (reality) is unknown, the error is also unknown and can only be statistically quantified—that is, the error is uncertain, but has a single unique (though unknowable) value. In practice, errors can be approximated using residuals—the amounts by which observations differ from some estimator of their true values. For example, we might use the sample mean as an estimator for the true (unknown) population mean. In EO, this introduces an additional complication, in that we are typically interested in spatio-temporal fields, over which the phenomenon of interest typically varies at all scales, from the molecular to the planetary [11]. Thus, when we talk about ‘the surface air temperature at a specific location and time’ we need to be clear on whether this means: (i) the temperature at precisely that point in space and time; (ii) some average over a specific time period at that point; (iii) an average over some finite spatial extent at a specific time; or (vi) an average over both space and time.

We also need to consider the response function of the observation instrument, i.e. what is it really measuring physically. Thus, when describing error, and thus uncertainty, and thus that aspect of quality it is necessary to describe what is defined as the true value, or reference value in reality (shown in figure 1) and in particular its spatial and temporal extent.

### (b) Data quality standards

The ISO suite of standards has been adopted by large numbers of EO data providers. ISO 19113 [12] defines quality principles, which are applied in ISO 19115 (geographic metadata [13]). There are also related works in the Guide to the Uncertainty in Measurement series [14]. The metadata records in the current GEOSS use the ISO 19115 data model and its companion XML encoding (ISO 19139 [15]). ISO 19138 [16] defines quality measures, but this is expected to be superseded by a new standard, ISO 19157 [17], which also supersedes ISO 19113.

The current ISO suite addresses the following quality aspects, which might be characterized as *producer data quality elements* because they are known by the data producer:

— *completeness*: presence and absence of features, their attributes and relationships;
— *logical consistency*: degree of adherence to logical rules of data structure, attribution and relationships;
— *positional accuracy*: accuracy of the position of features;
— *temporal accuracy*: accuracy of the temporal attributes and temporal relationships of features;
— *thematic accuracy*: accuracy of quantitative attributes and the correctness of non-quantitative attributes and of the classifications of features and their relationships; and
— *lineage*: information about the provenance of the dataset, including details of processing applied.

The first five of these are often referred to by researchers as the ‘famous five’ quality indicators for the evaluation of spatial data quality. As we shall see in this paper, we contend that these aspects, although valuable, are not entirely sufficient to meet user needs.

## 3. User needs for data quality

In general, a user may wish to see different aspects of data quality at different stages in his/her work. For example, in data discovery/search, users might be most interested in subjective statements about the utility of the dataset for a specific purpose, particularly from peers whom they trust [18]. They are less likely to be interested in detailed accuracy information per data item, because for large datasets this is likely to be very extensive and require summarization. However, they may well need to know that this more detailed information exists, and is reliable. This introduces the concept of the granularity, or scope, of the data quality descriptors, which is more extensively discussed in §5*d*.

When using the data, particularly for modelling or decision-making, quantified accuracy judgements are required in order to use the quality information in anything other than trivial ways. For example, it is impossible to propagate subjective textual statements of belief about data quality through a workflow. In this case, one can capture only the subjective judgements as metadata on the outputs, so that other users can form their own opinions about the quality of the outputs, based on their beliefs about the processing operations and inputs. However, if quantified quality (accuracy) information is available, then in theory it is possible to propagate this information through workflows in a principled manner to provide quantified quality (uncertainty) information on the outputs, using for example Monte Carlo methods [19]. We argue that to use such outputs in a rational decision-making framework [20] quantified quality information is crucial.

One aim of our research is to identify useful quality indicators for geospatial data which can be standardized to enable comparison of datasets against user requirements. To achieve that, we carried out a survey to assess the perceptions and requirements of real data users and producers. The survey questionnaire was carefully designed to concentrate on high-level questions about the interviewee's current area of work, usage and selection of external data sources. We purposely avoid using the term ‘quality’ in the questionnaire to avoid biasing the results with the users’ preconceived notions of the meaning of the term. We performed semi-structured face-to-face and telephone interviews in order to gain maximum value from the consultation process, rather than asking interviewees to fill in the questionnaire unsupervised.

We conducted a total of 18 interviews, going beyond a ‘data saturation point’—the point at which consulting further participants was unlikely to provide new information or themes in the data [21,22]. The data saturation point depends on the study conducted and may require as few as 12 participants to reveal most commonly occurring themes [21]. In our study, the data saturation point occurred after six interviews; so the additional consultations were mostly used for verification purposes. We selected a range of interviewees to include archivists, system architects, climate forecasters, land-use researchers, environmental researchers and academics. The data collected allowed us to elicit a variety of user stories and develop a wide-ranging picture of user needs, which led to the identification of a set of potential quality indicators. There is not sufficient space in this paper to examine the results of this consultation in detail; so we confine ourselves to outlining our general findings and expanding on two particular case studies.

### (a) General results of user consultation

Our analysis identified that geospatial data users are exceedingly interested in good quality metadata records. At present, users find that metadata records are typically incomplete with a lot of essential data omitted. Despite the work of the standardization bodies towards establishing core metadata elements and enforcing good metadata practices, dataset providers do not always follow standards, and may leave metadata records incomplete (see §4 for a more detailed study of this). This makes the process of dataset discovery and selection more difficult. The interviewed users and experts stated that core metadata defined in ISO and Dublin Core standards must be provided with the geospatial datasets to enable effective data quality evaluation.

Users are also interested in ‘soft’ knowledge about data quality—i.e. data providers’ comments on the overall quality of a dataset, any data errors, potential data use and any other information that can help to assess fitness-for-use of the data. Our interviewees stressed that sometimes such data quality measures cannot be recorded in standard metadata records, but would significantly help in evaluation of data quality and more effective data use.

Another very important quality indicator identified in our survey is peer recommendations and reviews. Geospatial data users and experts stated that they rely heavily on peer feedback and recommendations when selecting a dataset, and that they contact their peers to obtain suggestions on what datasets are most suitable and are of good quality.

Our study also revealed the importance of dataset provenance as well as citation and licensing information when assessing whether data are fit for purpose. Users confirmed that provenance information is usually incomplete, citation information is hard to acquire and licensing information is often missing from the metadata records of datasets. Experts stated that they typically require information about dataset providers, and, in particular, valid contact details. The reputation of data providers was identified as a key factor in dataset selection. Users typically rely on data from producers that they already know or those who have a good reputation in the community.

Our findings also indicate the importance of being able to inspect complex metadata rapidly and intuitively. In particular, our interviewees stressed the need for a mechanism to enable them to easily and systematically compare several metadata records. Quality information visualization would allow geospatial datasets to be compared more effectively, especially when datasets are very similar and differences are hard to distinguish. Such functionality (perhaps a side-by-side, like-for-like comparison) would support and simplify data searches, decision-making and data quality evaluation, particularly for less knowledgeable and non-expert users who find it hard to manually inspect data to assess their fitness-for-use. This use of comparative search and visualization techniques could apply to all types of quality information, from the dataset to the pixel level.

### (b) Case study 1: crop yield monitoring

**Figure 2.**
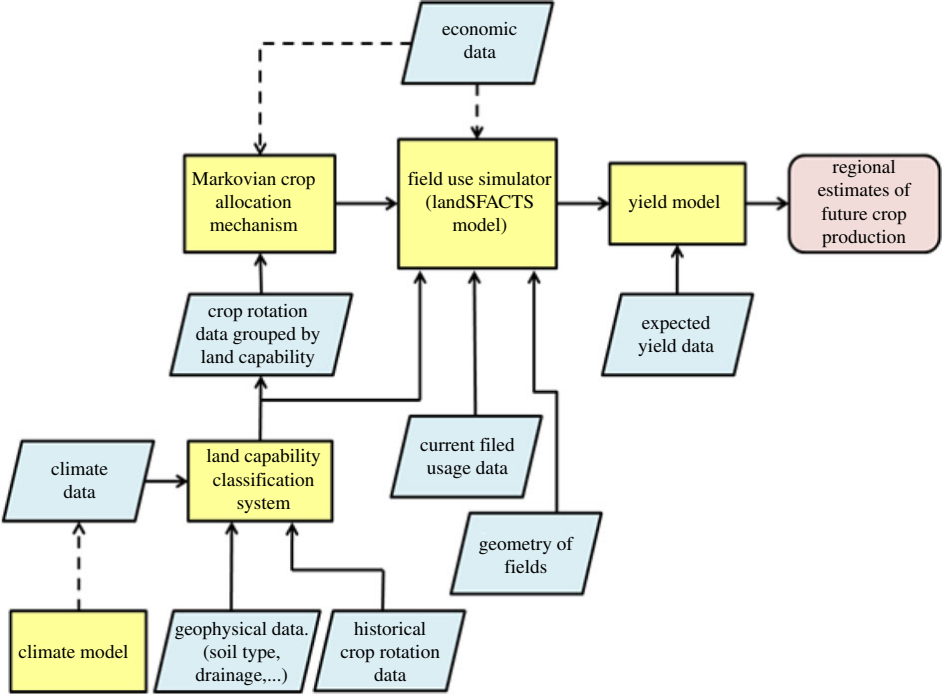
An illustration of the data (rhomboids) and model (rectangles) resources used to compute future regional estimates of crop yield. (Online version in colour.)

This case study was generated from consultation with the Food and Environment Research Agency. A scientist, Jane, is asked to assess potential changes in agricultural crop production in the UK, between 2010 and 2050. This raises several challenges, particularly regarding the uncertainty of information from global climate models, and their downscaling to field-level predictions. However, as this is an important policy issue, Jane decides to attempt to quantify those uncertainties on which she is able to make a judgement, and to build an overall conceptual model defining key component models and their data or parameter inputs. Jane identifies the need for an annual crop allocation model to predict possible UK crop growth patterns, which will require inputs describing the geophysical characteristics of individual fields, in terms of climate/weather and soil properties. This model also requires information about economic context and possibly about farmer behaviour. Fortunately, Jane has a significant amount of historical data for 1990–2000 which characterizes UK cropping patterns and rotations, and she is confident that these data have very few errors. Jane also requires a model to predict possible crop yields, but here we will focus only on the crop allocation modelling (figure 2).

Jane must identify sources of current, and future, climate data, and discover data about soils across the UK. She uses a probabilistic framework to construct her overall model, taking into account quantified uncertainty where possible. Jane believes this will help her convey the confidence in her answers more honestly and transparently, with each judgement quantified. Thus, for the discovery phase, Jane is most concerned to find data that have associated quality statements, including numerical estimates of uncertainty, ideally at the data item level (e.g. an error estimate for her specific spatio-temporal sample), rather than an overall assessment for the data product (e.g. compliance to calibration criteria). Jane wants the best data (in terms of minimal uncertainty with respect to reality) subject to some constraints on spatial and temporal coverage of the data, and their relevance to her modelling task. It is particularly challenging to quantify the uncertainties on global climate predictions downscaled to the level of individual fields. Here, Jane will explore judgements of data producers and other scientists on the quality of both the climate predictions and the uncertainty estimates supplied with them.

This case study shows that user needs at the discovery stage are heavily influenced by the planned use of the data (strategic policy modelling which propagates uncertainty), and, in order to assess fitness-for-purpose, appropriate quality information is critical, which can include per-product quality; availability and reliability of per-item quality information; granularity of per-item quality information (dataset, field or pixel); nature of per-item quality information (she requires quantified values); extent, completeness, legend, lineage and reputation of data; and community assessments of data relevance and usability within this application domain, etc.

### (c) Case study 2: ocean data reanalysis

This case study was generated from consultation with a scientist on the MyOcean project (http://www.myocean.eu), which aims to create a *reanalysis*, i.e. an estimate of the state of the past oceans using historical observations in conjunction with state-of-the-art numerical modelling. An important part of the reanalysis process is *validation*, in which the reanalysis results are compared with known and trusted datasets in order to characterize the quality of the reanalysis.

Decisions about which datasets to validate against are taken extremely carefully by the community based on criteria such as their heritage and use in previous projects, whether the datasets are published and supported by citations in the scientific literature, whether the datasets are published with error estimates, the spatial resolution of the data and the independence of the datasets from the data that were used in the reanalysis.

The last point is particularly noteworthy: it requires knowledge of the complete processing chain that was applied to both the reanalysis and the validation datasets. A search for independent data would therefore be quite complicated, e.g. ‘find me all datasets of sea surface temperature that were not produced using any of the datasets that were input to my reanalysis’.

This case study shows that users sometimes require extremely detailed information about the heritage of a dataset, encompassing producer-specified data (e.g. provenance and error estimates) and user-generated metadata (citations and comments).

## 4. Current state of the GEOSS quality metadata records

In order to analyse how well quality concepts are being communicated in EO and therefore how the situation can be improved, this section presents an exhaustive study of the producer data quality aspects available in the GEOSS clearinghouse metadata catalogue. The clearinghouse follows the Open Geospatial Consortium's Catalogue Services for the Web (CS-W [23]) standard and the metadata retrieved from it follows the standard ISO 19115 and is encoded in XML following ISO 19139 (see §2*b*). This allows the application of a semi-automatic methodology that was used in a previous study that analyses the quality of the metadata records (meta-quality) catalogued in Spanish regional spatial data infrastructures (SDIs [24]). In brief, the methodology consists of harvesting the metadata records using the CS-W protocol, and extracting the information into a database. Then, the data are analysed and results are summarized. In this case, the number of metadata records harvested was 97 203 (October 2011).

This section will focus on the results of the two main aspects emphasized in ISO 19115 as data quality information, directly related to the data quality information package (DQ_DataQuality). These are the quality elements (DQ_Element) and the lineage information (LI_Lineage). Moreover, we have also covered the usage information (MD_Usage). The overall number of metadata records with quality indicators is 19 107 (19.66%). These metadata records contain a total of 52 187 quality indicators, which represents a mean of 2.7 quality indicators per document. Table 1 represents the quality indicators classified according to the classes (generic quality indicators) and subclasses (specific quality indicators) established by ISO 19 115.

**Table 1.** Generic and specific quality indicators in the GEOSS clearinghouse.

generic quality indicator	specific quality indicator	MD records	percentage
positional accuracy	absolute external positional accuracy	17 767	34.04
	gridded data positional accuracy	1364	2.61
	relative internal positional accuracy	280	0.54
completeness	completeness commission	9815	18.81
	completeness omission	8823	16.91
logical consistency	conceptual consistency	9454	18.12
	domain consistency	857	1.64
	topological consistency	12	0.02
	format consistency	0	0
temporal accuracy	accuracy of a time measurement	2870	5.50
	temporal consistency	682	1.31
	temporal validity	0	0
thematic accuracy	non-quantitative attribute accuracy	2	0
	quantitative attribute accuracy	261	0.50
	thematic classification correctness	0	0
total quality indicators		52 187	100

The results show that, among the generic indicators, the most represented are positional accuracy and completeness, having a quite similar importance (36.65% and 35.72%, respectively) and reaching 72.37 per cent of the total. The five main quality indicators described in §2*a* are specialized in the 15 specific quality indicators; the most used is the absolute external positional accuracy, representing 34.04 per cent of the indicators. After that, we find completeness commission (18.81%), completeness omission (16.91%) and conceptual consistency (18.12%), reflecting a diversity in specific quality indicators in the metadata records. This result contrasts with a previous study about regional SDIs [24] where the completeness, the consistency and the temporal accuracy represent no more than 5 per cent overall, while, in the EO products analysed, these indicators represent 35.71 per cent, 19.78 per cent and 6.81 per cent, respectively.

A more detailed analysis can be done by classifying the quality indicators into quality measures (DQ_Results). In the overall metadata records, we find 25 944 quality measures. There are three types of measures: (i) numerical quantitative results (there are 22 275 such measures; 85.86%), (ii) specification conformance, in our case mainly to INSPIRE Directive (http://inspire.jrc.ec.europa.eu/) (3669 measures; 14.14%), and (iii) coverage result, conforming to the ISO 19115-2 standard (five measures; 0.02%), in which the quality is represented in an additional raster file. Unfortunately, the link to the file was missing at the time this study was performed.

Regarding the lineage (the last producer quality aspect in §2), ISO 19115 distinguishes between production processes (LI_ProcessStep) and the sources used in producing the dataset (LI_Source) that can be combined in several ways. Therefore, there are: (i) 3771 (3.88%) metadata records containing a direct list of the data sources, (ii) 9261 (9.53%) metadata records containing a direct list of the processes, and (iii) 1226 (1.26%) metadata records that link data sources to each process (complete provenance).

The usage (MD_Usage) is an entity part of the identification information package that is intended to identify the data and provides basic information about specific applications for which the resource has been or is being used by different users. Through this element, producers can describe the usages for which the dataset is created, or describe other applications that users have reported back to them. However, in practice, there is no satisfactory mechanism for users to record their feedback. There are 1133 (1.17%) records containing usage information, but only the mandatory specific usage and user contact information elements are described.

To summarize, these results show that quality indicators and lineage are far from complete in the GEOSS, but the current status is enough to start developing tools for exposing and exploiting the quality data that already exist. Even if some quality indicators are used, the name and description of the measure used to quantify the indicator are rarely well described, revealing the need for a good list of these measures. ISO 19115-2 extensions, which were created for imagery and other kinds of EO data, are rarely used in the GEOSS. Finally, the paucity of usage records is a clear indication that this mechanism is not the right solution for user feedback or is unknown by most of the users. Section 5 extends the ISO model to better address consumer quality aspects.

## 5. Improved quality information model

On the basis of the user consultations (§3) and the analysis of the current state of the GEOSS (§4), we propose an improved model for data quality information. This is based on existing standards (§2*b*) and is designed to be minimally invasive.

The new data model is split into two submodels, one for data quality information that will typically be generated by data producers (the ‘producer quality model’) and one for information that will typically be generated by users (the ‘user quality model’). Nothing prevents the information in the models being combined in a single system if appropriate for a given application. The models are under continuous development (under careful version control), and the current versions of both models at the time of writing are available as UML diagrams (see electronic supplementary material). (The diagrams are too large and complex to be included as figures in this paper.) From the UML diagrams, XML schemas have been automatically derived.

The main novel features of the model are discussed in the following sections. The model will be described in greater detail in future specialist publications.

### (a) User feedback

Many users commented that they come to trust data based upon studies performed by their peers, in addition to information provided by the original data provider (§3*c*). The primary requirement was for a means to link datasets with relevant citations in the scholarly literature, but a desire was also expressed for less formal feedback mechanisms such as user comments. Data providers have also expressed their desire for such a system, as user feedback is a key driver for them to improve their data products. (Note that ISO 19115 defines a ‘Citation’ class, but this is used to specify a mechanism for citing a dataset, not for linking to external citations *about* the dataset.) As described in §4, the MD_Usage class appears not to be a suitable or successful means to record user feedback information.

The user quality model includes a comprehensive set of mechanisms for recording feedback, including free-text comments (the GVQ_UserComment class), a numerical rating (GVQ_Rating), a publication in the literature (GVQ_Publication) or a discovered issue (GVQ_DiscoveredIssue). A ‘discovered issue’ records information about a problem with a dataset that the user has discovered, and which may be of interest to other users, together with information about suggested workarounds or alternative datasets.

### (b) Enhancements to data-producer capabilities

The existing ISO 19115 and 19157 standards contain some of the information classes that are needed by data producers to describe their datasets. Our new model adds the capability to record publications in the literature and discovered issues (using the same classes as the user quality model), together with an enhanced capability to record provenance information through specializations of the ISO LI_Lineage classes.

Additionally, a means is provided to characterize datasets against a reference standard, as recommended by the QA4EO guidelines. The new model specializes the ISO 19157 DQ_Evaluation classes, allowing reference datasets to be associated as MD_Associated Resource instances.

### (c) Describing quantitative uncertainties using UncertML

A particular issue with the existing ISO 19138 and superseding ISO 19157 standards is that the quality measures contained within the proposed standard do not, in themselves, provide a complete and self-consistent statistical definition of quantitative uncertainty. For example, a DQ_QuantitativeResult may be produced which conveys the vertical accuracy of a DEM, as follows:


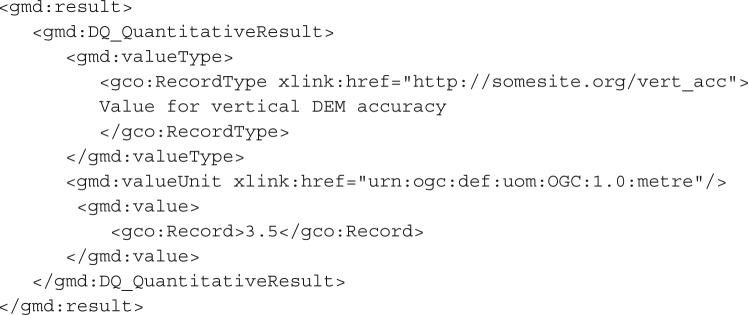


This description is fairly rich in information, provided that it is used by a reader who can understand the semantics. If it is contained within a DQ_PositionalAccuracy element, or one of the ‘credible interval’ elements provided by ISO 19138, then the potential for automatic use of the data is increased. However, this encoding is deficient in the statistical information we can obtain about the uncertainty itself. Was the spread of error at the sampled locations genuinely Gaussian? Was there any bias in the error? How many samples were taken in order to calculate this value? We could assume that the ‘3.5’ value here represents two standard deviations around a Gaussian mean, but even this information is not automatically apparent from the above example. The questions above are particularly important for propagating this uncertainty through a modelling process and adequately handling its impact.

We propose the use of the UncertML v. 2.0 dictionary and associated schema. UncertML is based on concretely defined elements, which represent a number of distributions, summary statistics, realizations and samples. An example of an UncertML XML fragment element (representing an interquartile range) is shown below—more may be seen in the user guide at http://www.uncertml.org:


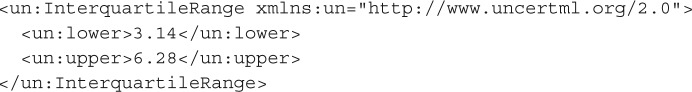


This brings us to the important question: should uncertainty be considered as metadata, or is it more realistic to recognize the inherent uncertainty of all data and to treat the uncertain values (whether it be a distribution, an interval or a sample) as the data themselves? This philosophical issue has practical impact when considering encoding semantics such as how an UncertML ‘Uncertainty’ element should relate to existing quality elements such as the ‘DQ_QuantitativeResult’. The latter approach would mean that an UncertML ‘Uncertainty’ element is actually a realization of the ‘DQ_QuantitativeResult’. However, this causes complications owing to the design of UncertML, which separates concerns and ensures that UncertML does not represent phenomena outside the remit for which it should be complete: the probabilistic representation of uncertainty. For this reason, units of measure (required for the ‘ValueUnit’ element of the DQ_QuantitativeResult) and other phenomenological information are not included in UncertML, but must be handled by an existing and appropriate schema. A more logical approach which is minimally invasive is to give an UncertML dictionary URI as the ‘valueType’. The example below takes this approach to encode exactly the same information as in the first example in this section, assuming that the errors have a mean of 0 (i.e. no bias):


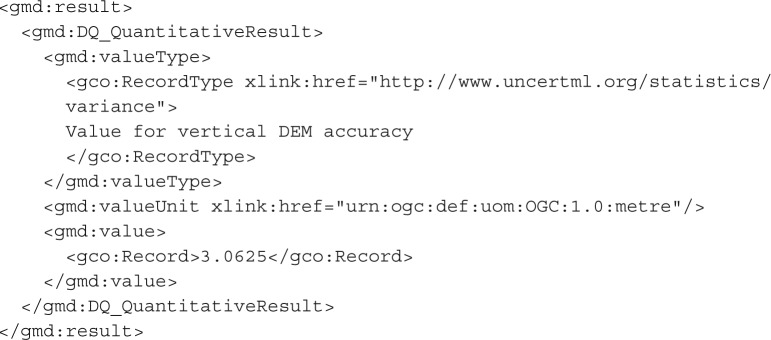


If the user is prepared to embed an UncertML ‘Uncertainty’ element as the value of their ‘DQ_Element’, even richer information can be conveyed, as follows:


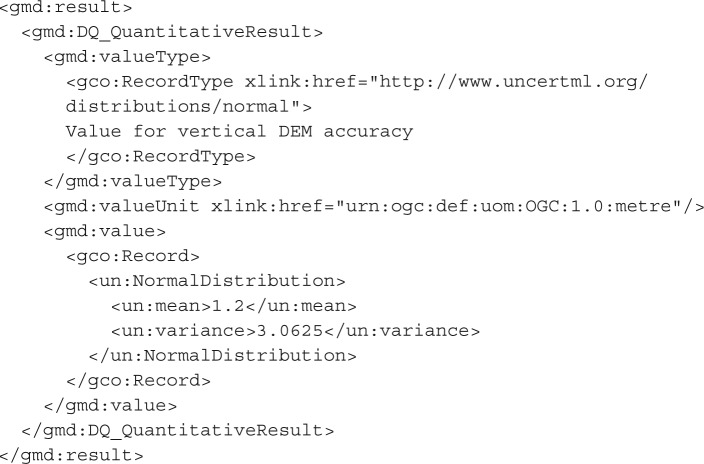


In the above example, the distribution of values from which the vertical accuracy was calculated is given in the form of a ‘un:NormalDistribution’ element. This time, there is an explicit recognition that the errors were considered to acceptably fit a normal distribution with mean 1.2 (i.e. an overall positive bias was observed—a difficult feature to convey by traditional means) and a publicly accessible dictionary definition of the distribution is referenced.

### (d) Hierarchical treatment of metadata

Examination of typical spatial data holdings reveals a technical requirement in addition to the user requirements discussed earlier. Many datasets are very large and hierarchical in nature. Quality information may apply at different levels in the hierarchy: for example, in an EO scenario, quality information can be given for a whole platform series (e.g. the Landsat-5 [25] image series), for a single dataset (e.g. a specific scene of a Landsat-5 image), for a single band (e.g. the near infrared Landsat-5 band) or even for a specific pixel of the image.

Storing all metadata at all levels is impractical and would lead to redundancy, inconsistency or incompleteness of metadata. We propose an inheritance mechanism to store each metadata item and quality information at the optimum hierarchical level and to allow an easy and efficient documentation of metadata. This applies not only to quality metadata but to many other metadata types as well, and is applicable to many kinds of geographical data from topographic maps to multi-band satellite imagery.

**Figure 3.**
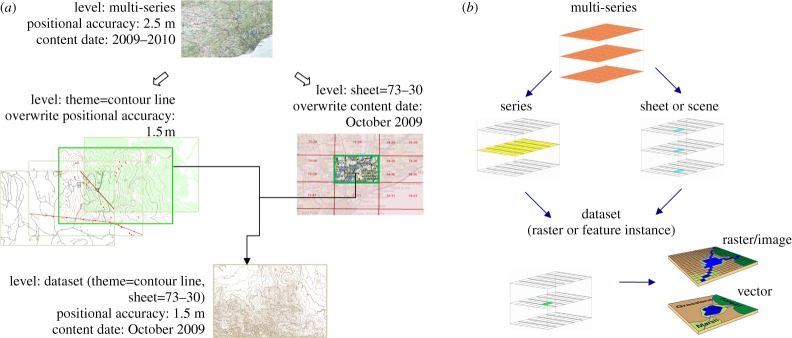
(*a*) Topographic map as a whole, including several layers from the thematic point of view that are split into tiles. (*b*) Schematic representation of hierarchy levels defined in the model. (Online version in colour.)

Several hierarchy levels can be defined on the model: multi-series, series, topographic sheet or EO scene, dataset and feature or pixel instance, as shown in figure 3*b*. Metadata items are documented only for the highest applicable level and then are automatically inherited by all the dependent elements.

ISO 19115 suggests that metadata aggregated levels can be defined so that a generic metadata record for an aggregation is inherited by a child dataset but can be overwritten using specific values. We propose a more complete approach in Zabala & Masó [26] that extends ISO 19115 to define several upper level aggregations and several inheritance types depending on the metadata elements to which they apply:

— *No inheritance.* Some metadata elements make sense on all the hierarchy levels and are never inherited; for example, fileIdentifier, hierarchyLevel, dateStamp.
— *Extensible inheritance.* Metadata elements with multiple cardinality allowing general values to be inherited from upper levels as well as defining particular values at lower levels that are added to the previous ones, e.g. alternateTitle, metadata contact, publication and citation and user feedback (the last two are from the extended quality model in §5).
— *Specifiable inheritance.* Metadata elements that if defined on upper levels must be adopted or specified by lower levels but cannot be left undefined, e.g. quality elements, equivalent scale, discovered issues (from the extended quality model).
— *Combined inheritance.* Typically for free-text metadata elements, a concatenation method has been designed allowing the metadata value at a specific hierarchy level to be defined combining the values of the same metadata element at upper levels following a pattern and allowing some additional information also to be defined, e.g. the title, lineage statement, etc.

This hierarchical metadata treatment has been applied to the 1 : 25 000 topographical map downloaded from the Catalan Cartographic Institute (ICC) (http://www.icc.cat), which covers several thematic layers and is tiled on topographic sheets in order to obtain manageable files. Instead of including the quality indicators for each individual dataset in the series, some generic ones can be selected (e.g. conceptual consistency to ICC specifications) and documented only once at the multi-series level, keeping the specifics only at the theme level (e.g. thematic accuracy) and at sheet level (e.g. temporal consistency) or even at the dataset level if needed. The metadata following the hierarchical model avoid repetition and increase metadata coherence among all individual datasets in the series. Once implemented, we checked that repeated queries performed by users returned complete metadata reports for each dataset that integrate the metadata quality coming from different levels. By including information about the accuracy of individual pixels in a DEM, a flood risk map could be derived.

The hierarchical metadata model is readily applicable to the new quality model proposed in this paper. For example, the overall positional accuracy of a satellite product can be defined for nearly all satellite data-processing levels on a scene because they are derived from the same geometric correction process done early in the processing chain. On the other hand, the overall thematic accuracy of a specific derived product can be defined for the whole set of scenes because they are obtained in the same way (e.g. using the same instrument). For a specific product and scene, a specific value for either overall thematic or positional accuracy can be defined. Furthermore, these quality parameters can be even provided for each pixel (e.g. by a specific per-pixel quality flag that marks pixels with clouds). Extended elements also use those inheritance rules; for example, a journal paper citing the whole Landsat mission is defined on the ‘publication and citation’ element at multi-series level and inherited by all the lower levels (products and scenes). Other levels can add a citation of a specific publication focused on a scene of a product.

One limitation of this model is that there is no clear way to associate a quality element to a specific wavelength band in a remotely sensed image (the MD_ScopeCode list has no ‘band’ entry). Possible alternatives could be to assimilate the ‘attributeType’ to band (ISO 19115-1 already names the role of MD_RangeDimension aggregation as ‘attribute’) or to include a new DQ_DataQuality element in the MD_RangeDimension definition.

## 6. Discussion and future work

The innovations in informatics described in this paper are in the process of being implemented in prototype software within GeoViQua and related projects. For example, the GEOSSBack portal (http://www.ogc.uab.es/GEOSSBack) is a system that combines GEOSS search capabilities with a feedback interface that allows users to comment on datasets. The GEOSSBack portal can allow metadata about a particular topic to be searched and a collection of related datasets identified. The user can pick a particular dataset and read the catalogued metadata from the producer and also the previous user comments. Additionally, the user can enter new comments or update their own. All of this is stored and becomes immediately available to other users. More work will be required to evaluate and evolve this system.

A software tool has been created to take quality metadata recorded in the XML format defined from the UML diagrams described in §5 and automatically generate comprehensive metadata reports as Web pages. These reports include a great deal of precise information about data quality in a human-readable format, and provide a means for users to provide feedback on the efficacy of the quality model without the need to understand the technical details of the information model. Future studies will examine the users’ reaction to these reports and may lead to refinement of the way the information is structured and presented. A sample report is provided in the electronic supplementary material.

We noted in §3 that a means to visualize quality information is required, either as side-by-side metadata records or as visualizations of uncertainty estimates. The GeoViQua project is actively researching these methods, which will be the subject of future publications. The modified information model described in this paper will be a key component of this, allowing consistent recording of quality information. Mechanisms for visualization of data quality in the Open Geospatial Consortium technologies (notably the Web Map Service [27] and the KML data format [28]) will be developed.

Strongly related to data quality is the complete capture of origins of quality information (meta-quality, as defined in ISO 19157). Meta-quality, as the name suggests, is information about the quality of the quality information [29]. Particular aspects require further investigation in the quality model to support meta-quality, including:

— providing a mechanism to represent the lineage of quality information;
— supporting this with a link to the reference data used in the quality assessment;
— accompanying the quality metadata with producer caveats on applicability and reliability of the quality information; and
— supporting user feedback on the data quality statements themselves, not just on the data.

The provision of meta-quality will work to further engender trust in users of the data, because it is essential that users can also trust the quality information itself. Clearly, there is the potential for an infinite hierarchy of meta-meta-quality; however, we believe that meta-quality would be most usefully provided in a form that describes as completely as possible how the quality indicators were arrived at, so that they could potentially be reproduced. This answers the QA4EO guidelines (see §1), which state that quality indicators must be published alongside their derivation.

## 7. Conclusions

In this study, we addressed the problem of EO data quality from a number of different angles, including (i) clarifying concepts and terminology, (ii) reviewing existing relevant international standards, (iii) establishing unfulfilled user needs, and (iv) surveying the current state of the GEOSS. We proposed an expanded model for representation of EO data quality and described how it can be applied to large data holdings through a metadata inheritance mechanism.

Although specifically aimed at the EO community, this study is relevant to many other areas of e-Science, particularly where data need to be provided to a diverse user community. Many of the concepts (e.g. UncertML) are entirely general and can be much more widely applied. Conversely, the EO informatics community will benefit from innovations occurring in other communities, particularly concerning the linking of information from different sources. We note that, for these linkages to be effective, GEOSS requires a robust mechanism for globally, uniquely and permanently identifying datasets, something that is currently lacking. A strong need was also identified to help users to understand the provenance of datasets. The W3C provides a generic provenance model for data on the Internet [30], and there is an almost one-to-one correspondence between this model and the ISO 19115 Lineage model. There is therefore strong potential for interoperability with other communities.
